# Strategies to improve delivery of primary eye care in public health care institutions in Sri Lanka

**Published:** 2022-03-01

**Authors:** Prabhath Piyasena, Vindya Kumarapeli, Dhanushka Abeygunathilaka

**Affiliations:** 1 Medical Officer: Directorate of Policy Analysis and Development, Ministry of Health, Sri Lanka.; 2 Acting Director: Directorate of Policy Analysis and Development, Ministry of Health, Sri Lanka.; 3 Specialist Registrar in Community Medicine: Directorate of Non-Communicable Diseases, Ministry of Health, Sri Lanka.

**Primary eye care is integrated within the public health care system by providing primary eye care at the outpatient departments of secondary and tertiary heath care institutions, with unrestricted access.**

Despite a strong primary health care system in Sri Lanka, the successful integration of eye care services at the primary level of health service delivery is still evolving. The majority of eye care service provision is at the secondary and tertiary levels.

According to a study of blindness and visual impairment in Sri Lanka that examined a nationally representative sample of 5,779 people aged ≥ 40 years, the prevalence of blindness in the country is 1.7% and that of low vision is 17%. Cataract (66.7%) and uncorrected refractive errors (12.5%) are the most common causes of blindness.^1^ Blindness is significantly higher in those aged ≥ 70 years, as compared to those aged 40 to 49 years.^1^ The data are helpful for assessing and planning for the eye care needs of the population. The focus on improving service delivery at the primary eye care level is an important step in identifying the needs early.

## Government policy on primary health care

The Sri Lanka National Health Policy 2016–2025 endorses a people-centred health system based on universal health coverage.^2^ The National Health Policy builds on the mandates of quality health care and equitable coverage, which are the focus points of the 2018 policy on health care delivery for universal health.^3^ The provision of first contact care through the strengthening of the primary care system has been given top priority. In this strategic approach, primary eye care is integrated within the primary health care system.

**Figure:**
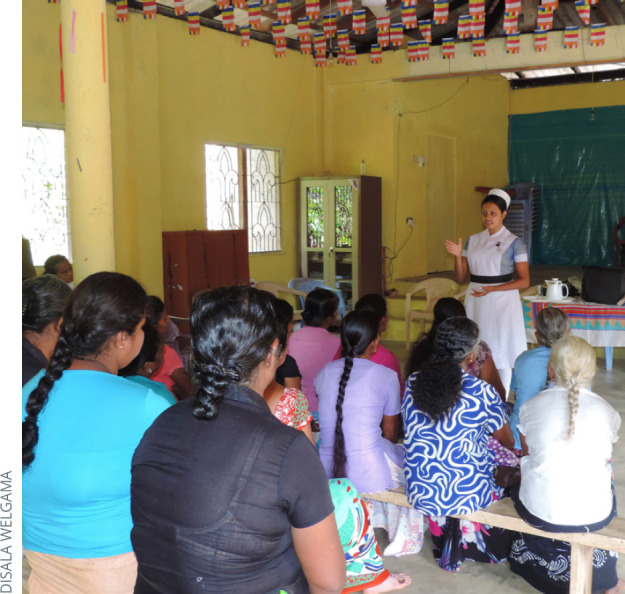
Health education by a nursing officer at a primary care clinic in Palmadulla. **SRI LANKA.**

The provincial health ministries manage the primary health care (PHC) institutions in the nine administrative divisions, or provinces, in Sri Lanka.^3^ The Sri Lankan government aims to provide health services free of charge at the point of delivery and spends 3.24% of gross domestic product on health. The organisational structure consists of 1,067 public sector heath care facilities: all tertiary (22) and most of the secondary level (94) health care institutions under the ministry of health centrally; and several secondary and all primary level facilities (470 divisional hospitals and 475 primary care units) under the provincial councils at the sub-national level, give access to a health care facility within a 4.8 km range in any region. One primary care institution covers about 5,000 people at the community level. The public sector provides

85 to 90% of the inpatient care. Sri Lanka delivers primary health care, inclusive of eye care, through two models: preventive services and curative interventions.^4^

## Primary eye care service delivery

Primary health care delivery is through a range of health care facilities: central dispensaries, peripheral units, and divisional hospitals—all designated as primary care medical institutions (PMCIs)—which are equipped to provide basic eye care, such as treating chalazion and conjunctivitis, screening for visual acuity to identify refractive errors and cataract, and referring patients for further care at a specialist eye clinic at the secondary or tertiary level. The new essential services package strategy aims to strengthen the delivery of primary eye care services at primary care medical institutions by improving the availability of medical supplies and equipment. A qualified medical officer provides services in most of these institutions; however, there is no specific ophthalmic training for these medical officers at present other than any training they may have obtained optionally under the basic medical degree.

A key feature of the current health system is that strategies are being identified and developed for the integration of primary eye care service delivery with the outpatient departments of secondary and tertiary level health care institutions, with unrestricted access and no charge.

**Figure 1:**
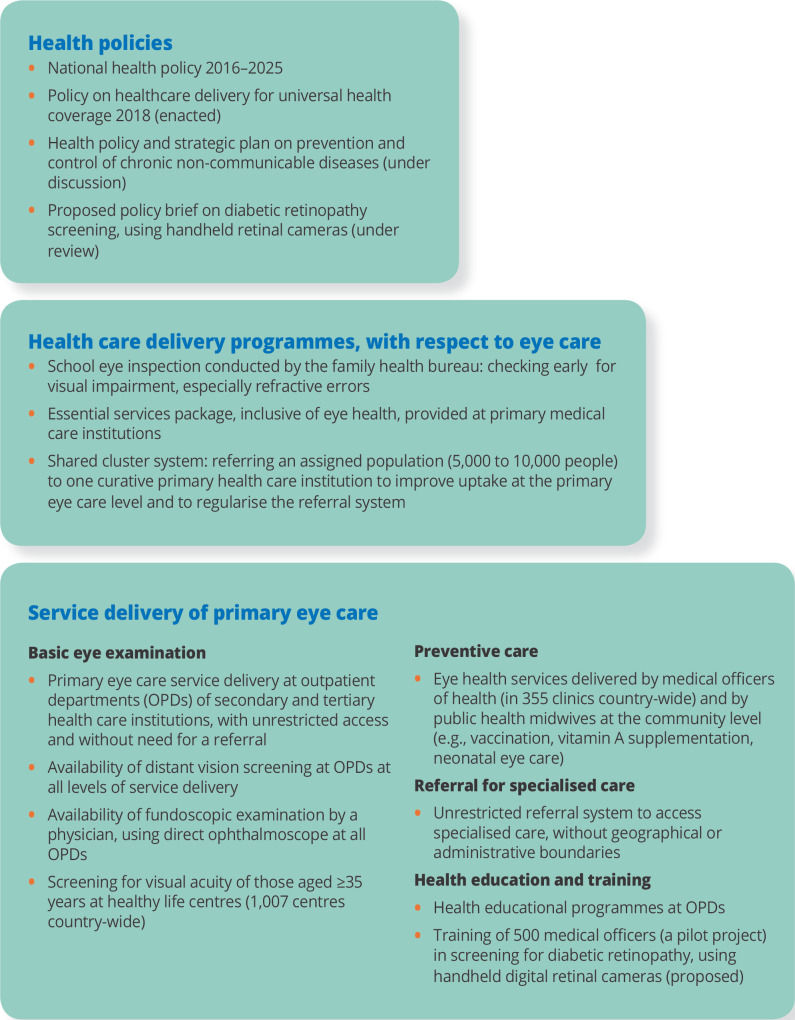
Integrating primary eye care with public health care

Figure 1 describes the existing policies and programmes, service packages, and delivery models with respect to primary eye care.

## Way forward

Although Sri Lanka has an exemplary primary health care service network, it needs to strengthen the delivery of primary eye care services. A shortage of appropriately skilled mid-level eye care personnel and a lack of medical supplies and technology have been identified as the main barriers.^5^ The current unrestricted referral system is leading to overcrowding in specialised clinics at the secondary and tertiary levels.

Service delivery that can meet individual and population needs, effective training modules for mid-level eye health personnel, multisectoral collaboration, community participation, and the development of infrastructure in rural areas to bridge disparities are essential strategies to serve the country's evolving eye health needs.^6^^,^^7^ There is a high potential for a successful blindness prevention programme in Sri Lanka, based on systems established under the VISION 2020 country programme in previous years.
